# Phacomatosis cesio-flammeo-marmorata: report of a rare association^☆^

**DOI:** 10.1016/j.abd.2022.09.019

**Published:** 2024-01-17

**Authors:** Luciane Zagonel, Mônica Vannucci Nunes Lipay, Glaucos Ricardo Paraluppi, Célia Antônia Xavier de Moraes Alves

**Affiliations:** Department of Dermatology, Faculty of Medicine of Jundiaí, Jundiaí, SP, Brazil

Dear Editor,

Phacomatosis pigmentovascularis (PPV) is a rare congenital anomaly characterized by the presence of vascular and pigmentary lesions. The present case describes a rare association of congenital telangiectatic cutis marmorata, nevus flammeus and aberrant mongolian spots. These findings do not allow it to be classified under any item of the classification of PPV, except for the proposed name phacomatosis cesio-flammeo-marmorata.^1^

A late preterm baby was born from a dichorionic and diamniotic twin pregnancy to non-consanguineous parents; the mother had SARS-CoV-2 during an otherwise uneventful pregnancy and childbirth. The twin brother presented no alterations. Since birth, the reported brother however showed an erythematous lesion affecting almost the entire facial region, compatible with nevus flammeus. There was also a reticular pattern erythema on the right side of the body with clear demarcation in the anterior midline, compatible with cutis marmorata telangiectatica congenita, and extensive blue-gray macules on the lower limbs, gluteal area and back, compatible with mongolian spots. He also had grayish sclerae bilaterally and a slight asymmetry in the circumference of the lower limbs. The vascular lesions became more intense while crying or after exposure to cold (Figure 1, Figure 2).

**Figure 1:**
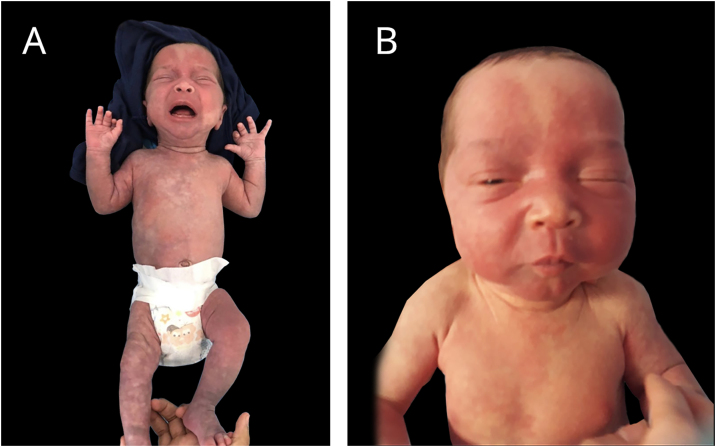
A and B: At 28 days of life, congenital telangiectatic cutis, aberrant mongolian spots and nevus flammeus on the face.

**Figure 2:**
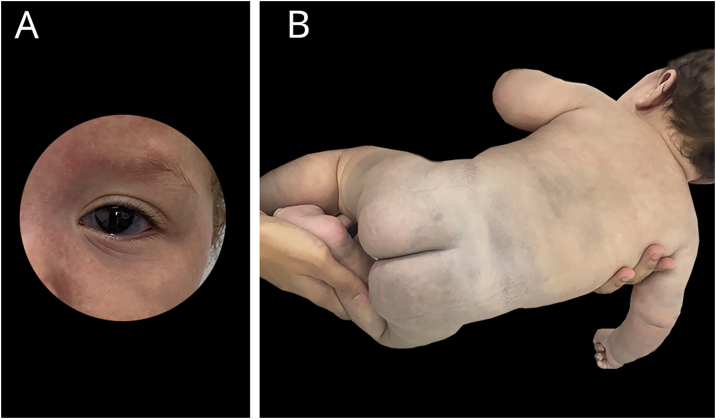
A and B: At six months of life, extensive mongolian spots and gray sclerae.

An echocardiogram and transfontanellar, and abdominal ultrasonography were performed, which showed no alterations. The neurological, ophthalmic, and orthopedic evaluations showed no abnormalities. Dermatological ultrasonography identified subcutaneous tissue hypoplasia in the right leg compared to the contralateral limb.

The whole exome sequencing in peripheral blood identified a probably pathogenic heterozygous variant in the COL17A1 gene, (c.3277+1G>A), in addition to a pathogenic heterozygous variant in the HBB gene, (c.20A>T: p., Glu7Val) attributing betalassemia minor.

At the time of this publication, the patient is one year old, showing normal neuropsychomotor development and partial improvement of the vascular lesions. The diagnosis of phacomatosis pigmentovascularis was made; however, the findings do not completely align with the current criteria, which made the classification challenging. The name phacomatosis cesio-flammeo-marmorata has already been proposed in the literature, with seven cases identified to date,^1^ which show heterogeneity of characteristics and one case with the described triad associated with nevus of Ota and anemic nevus.^2^

## Phacomatosis

PPV is a group of rare congenital syndromes characterized by the combination of capillary malformations and pigmented lesions, associated or not with systemic manifestations. Initially, it was classified into four types according to the phenotype (I - Nevus flammeus and nevus pigmentosus or verrucous; II - flammeus +/- anemic and mongolian spots; III - flammeus +/- anemic and spilus; IV - flammeus +/- anemic and spilus with mongolian spot) and subtypes (a –cutaneous involvement only; b – systemic involvement). Happle^3^ reclassified it into phacomatosis cesioflamea, cesiomarmorata, spilorosea, and unclassified, with or without systemic manifestations.

The syndrome pathogenesis is not well understood. The GNAG and GNA11 genes have been associated with some cases.^4^ The COL17A1 gene, identified in the patient of the present case, encodes collagen XVII, essential in epidermal stabilization, and, to the best of the authors knowledge, this is the first time that possibly pathogenic variants have been identified in this gene in a patient with these characteristics. This gene is related to epidermolysis bullosa; however, it does not have 100% penetrance, which may explain the lack of the expected phenotype. The patient does not have skin fragility so far.

The diagnosis of PPV is clinical; however, the classification is challenging when it does not meet all the criteria or if there is an overlap between them, which is why it has been proposed that the combination of the three conditions found in this patient merit a distinct classification as phacomatosis cesio-flammeo-marmorata.^1^ The investigation of ocular and neurological involvement is generally recommended,^5^ as well as other tests, depending on clinical suspicion.

## Financial support

None declared.

## Authors’ contributions

Luciane Zagonel: Approval of the final version of the manuscript; drafting and editing of the manuscript; critical review of the literature.

Mônica Vannucci Nunes Lipay: Approval of the final version of the manuscript; drafting and editing of the manuscript; critical review of the literature.

Glaucos Ricardo Paraluppi: Approval of the final version of the manuscript; critical review of the manuscript; intellectual participation in the propaedeutic and/or therapeutic conduct of the studied case.

Célia Antônia Xavier de Moraes Alves: Approval of the final version of the manuscript; critical review of the manuscript; intellectual participation in the propaedeutic and/or therapeutic conduct of the studied case.

## Conflicts of interest

None declared.
